# Individual differences in empathy‐related responses in early childhood: A person‐centred approach

**DOI:** 10.1111/bjdp.70042

**Published:** 2026-03-26

**Authors:** Johannes Bullinger, Natalie Christner, Radu Urian, Christina M. Kellermann, Seleste Beaulieu, Nikolaus Steinbeis, Kristen A. Dunfield, Markus Paulus

**Affiliations:** ^1^ Department of Psychology Ludwig‐Maximilians‐Universität München Munich Germany; ^2^ Department of Psychology Concordia University Montreal Quebec Canada; ^3^ Division of Psychology and Language Sciences University College London London UK

**Keywords:** empathy, person‐centred approach, preschool children, prosocial behaviour, social cognition

## Abstract

Empathy is vital for social development in early childhood. This study investigates the empathy‐related behaviours of 362 German and Canadian children (49.72% girls) aged 3 to 6 years (*M* = 60.65 months; *SD* = 11.43 months), focusing on responses to emotional distress. Using latent profile analysis, a person‐centred approach, we identified four profiles with distinct empathy‐related response patterns: prosocial, empathic, self‐distressed and uninvolved. To further delineate these profiles, we examined associations between profile membership and social–cognitive and social–emotional competencies. Results suggest that temperamental surgency and moral self‐concept differed across profiles. Self‐distressed children were rated lower in surgency compared with other profiles, and prosocial children had higher moral self‐concept than uninvolved children. Descriptively, profiles differed in emotion comprehension, with no significant post‐hoc differences, whereas Theory of Mind and inhibitory control did not differ between profiles. These results provide novel insights into the complex interplay of factors associated with children's reactions to emotional distress, enhancing our understanding of empathy development and informing future research directions.

## BACKGROUND

Empathy is the ability to understand and share someone else's emotional states while maintaining a clear distinction between self and others (Spinrad & Eisenberg, 2019). Empathy is crucial for moral development, as it motivates prosocial action and moral reasoning (Batson et al., 1986; Hoffman, 2000; Paulus, 2018). While debates continue about when these capacities first emerge (Paulus et al., 2024; Roth‐Hanania et al., 2011), empathic concern and comforting behaviours are clearly observable by age 3 (Dunfield & Kuhlmeier, 2013; Shoshani, 2024). The pre‐school period is particularly important for examining empathy because pre‐schoolers, unlike toddlers, demonstrate the self‐other differentiation and emotion regulation capacities necessary for examining individual differences in empathy‐related responses (Eisenberg et al., 2013; Hoffman, 2000). These responses vary considerably across children: While adequate self‐other differentiation enables empathic concern (also termed ‘sympathy’; Eisenberg et al., 1991; Malti et al., 2013), which motivates prosocial behaviour based on compassion for the victim (Hoffman, 2000), some children experience egoistic drift, becoming overwhelmed by personal distress and turning attention away from the victim. Pre‐schoolers thus exhibit diverse empathy‐related responses when observing emotional distress, each with distinct cognitive and emotional components (Eisenberg et al., 2015; Hoffman, 2000). The present study uses a person‐centred approach to identify distinct empathy‐related response profiles in children aged 3.5 to 6 years and examines how these profiles relate to Theory of Mind (ToM), emotion comprehension, moral self‐concept (MSC), inhibitory control and temperamental surgency. Identifying which factors distinguish children who effectively comfort others from those who become overwhelmed or disengaged has critical implications for supporting early moral development.

### Diverse empathy‐related responses to emotional distress

Hoffman's (2000) theory of empathy development provides a framework for understanding the diverse ways children respond to others' distress. As children construct a separate sense of self and other, their initial empathic distress can transform into empathic concern, that is compassion for the victim that motivates comforting. However, this transformation is not universal: Some children experience empathic over‐arousal, leading to egoistic drift where they become overwhelmed by personal distress and shift attention away from the victim. These individual differences manifest in distinct patterns of cognitive and emotional responses when observing others' distress.

The ways in which individuals respond when observing other's emotional distress are characterized by different cognitive and emotional patterns that lead to a diversity of empathy‐related responses. As a cognitive response, hypothesis testing describes attempts to understand the cause of another's emotional distress, ranging from verbal exploration (e.g. ‘Why are you sad?’) to physical approaching and examining the situation (Zahn‐Waxler et al., 1992). This understanding further supports the ongoing development of distinguishing between one's own emotions and those of others (Decety & Svetlova, 2012). As children gain knowledge about the causes and correlates of emotions through hypothesis testing, they move beyond egocentric assumptions about what the other person needs, enabling them to display empathic concern more appropriately (Hoffman, 2000). Hypothesis testing increases with age across the second year of life (Zahn‐Waxler et al., 1992). Building on this cognitive foundation empathic concern is an other‐oriented affective aspect of empathy (Eisenberg et al., 1991). Observable indicators include worried facial expressions and concerned vocalizations (e.g. ‘Oh no!’; Zahn‐Waxler et al., 1992). Empathic concern increases from the toddler through the pre‐school years (Knafo et al., 2008) and shows individual variation in developmental trajectories through middle childhood (Kienbaum, 2014; Malti et al., 2013). Empathic concern motivates comforting behaviours (Paulus, 2018): When children feel compassion for another's distress, they help alleviate it through physical comfort (e.g. hugs, pats) or verbal reassurance (‘It's okay’). Children engage in intentional comforting, with increasing sophistication and situational appropriateness throughout the pre‐school years (Hoffman, 2000). Comforting behaviours emerge between ages 1 and 2 (Zahn‐Waxler et al., 1992), increasing in frequency and variety between ages 2 and 4 (Dunfield & Kuhlmeier, 2013; Gibhardt et al., 2024). In contrast to these other‐oriented responses, self‐distress is a self‐focused, aversive reaction, involving primary concern with alleviating one's own negative feelings rather than focusing on the distressed other (Decety & Svetlova, 2012; Eisenberg & Eggum, 2009). Observable behaviours include self‐soothing (thumb‐sucking, rocking), displays of fear or anxiety, crying in response to another's distress and freezing or appearing overwhelmed (Zahn‐Waxler et al., 1992). This response exemplifies Hoffman's (2000) concept of egoistic drift, where empathic over‐arousal overwhelms the child's regulatory capacities, preventing the cognitive and emotional resources necessary for other‐oriented comforting behaviours. Self‐distress becomes less frequent with age as self‐other differentiation and emotion regulation develop, enabling empathic concern to emerge more readily (Decety & Svetlova, 2012). Avoidance is another form of self‐focused responding and involves disengagement from others' negative emotional states, such as turning away, leaving the scene, maintaining focus on object play without looking at the suffering other or actively ignoring distress cues (Stifter & Braungart, 1995). Comforting requires emotional attunement and willingness to connect with the distressed person's emotional experience (Batson et al., 1986), which is challenging when avoidance of aversive emotions is a pursued strategy. Avoidance prevents children from practicing the skills needed to comfort others in distress and may reflect either a strategy to escape the emotional cost of empathic arousal or a failure of the distress cues to activate empathic responses.

Importantly, these developmental trends have largely been identified by employing variable‐centred approaches to examine empathy in children. Similarly, much of what we know about associated mechanisms relies on similar approaches. For example, overall, children's efforts to understand distress support their ability to feel genuine concern for the victim (Knafo et al., 2009). Relatedly, empathic concern is positively related to comforting (Shoshani, 2024; Williams et al., 2014), whereas self‐distress is negatively related to this prosocial behaviour (Fabes et al., 1994). However, these variable‐centred approaches face important limitations. First, they have focused on infants and toddlers (for an overview, see Davidov et al., 2025), with less attention to the pre‐school period when individual differences in empathy‐related response patterns become increasingly pronounced as children construct more sophisticated cognitive and regulatory capacities (Hoffman, 2000; Spinrad & Eisenberg, 2019). Second, and more fundamentally, variable‐centred approaches have advanced our understanding of how empathy‐related responses relate to one another on average, but they cannot identify distinct subgroups of children who exhibit qualitatively different within‐person configurations.

### A person‐centred approach of empathy‐related response patterns

Person‐centred approaches complement variable‐centred methods by identifying subgroups of children who show similar patterns across multiple variables simultaneously (Weller et al., 2020). This is particularly relevant for empathy‐related responses: Two children with identical levels of empathic concern may differ fundamentally, with one translating concern into effective comforting and the other becoming overwhelmed by self‐distress and disengaging from the victim. Variable‐centred approaches would treat these children as equivalent, whereas person‐centred analyses can identify such qualitatively distinct response configurations.

This study employs Latent Profile Analysis (LPA) to examine how hypothesis testing, empathic concern, comforting, self‐distress and avoidance cluster together within individuals to form distinct response profiles. Recent person‐centred research has successfully identified meaningful prosocial profiles in toddlers and pre‐schoolers. Three profiles were identified in 18‐month‐olds (no prosocial behaviour, moderate prosocial behaviour and frequent instrumental helpers), with maternal sensitivity and mental state language predicting profile membership (Newton et al., 2016). Another study identified three profiles in 18‐month‐olds (low prosocial, moderate prosocial and frequent helpers) and four profiles in children aged 4.5–6 years (adding a high prosocial group), demonstrating that children show consistency in prosocial responding across different tasks and over time (Schachner et al., 2018). Similarly, three profiles were found at 22 months (high prosocial, instrumental helper and low prosocial) and two profiles at 34 months (high prosocial and instrumental/empathic helpers), with temperamental impulsivity predicting profile membership (Song et al., 2023). These studies indicate that individual differences form distinct profiles of prosocial behaviour. However, no study has yet applied a person‐centred approach to examine profiles of empathy, including both other‐oriented responses (i.e. hypothesis testing, empathic concern, comforting) and self‐focused responses (i.e. self‐distress, avoidance), in pre‐school‐aged children.

### Unique characteristics of different behavioural profiles

Statistically deriving profiles is only the first step; equally important is understanding what characterizes children in different profiles. If distinct empathy‐related response profiles emerge, examining their correlates reveals the cognitive, affective and temperamental factors that support or constrain different patterns of responding to others' distress. Examining empathy‐related response profiles in pre‐schoolers (ages 3–6) rather than toddlers enables investigation of how sophisticated cognitive abilities such as ToM and emotion comprehension distinguish different response patterns, beyond temperamental or parenting factors (Newton et al., 2016; Song et al., 2023). We examine whether ToM, emotion comprehension, inhibitory control, MSC and temperamental surgency distinguish profiles.

ToM, the ability to recognize that others possess thoughts, beliefs, desires and emotions distinct from one's own (Wellman & Liu, 2004), is a socio‐cognitive skill that contributes to empathy and comforting development (Decety & Svetlova, 2012). Empirical and meta‐analytical evidence supports the relation between ToM proficiency and prosocial behaviour including comforting (Imuta et al., 2016). Emotion comprehension, the ability to recognize and understand others' emotions, enables more sophisticated empathic expressions and context‐appropriate responses as it develops (Decety, 2010). Indeed, children aged 3 to 6 with low emotion comprehension exhibited less self‐initiated prosocial behaviour (Knafo et al., 2011). Longitudinal research emphasizes emotion comprehension's relation to prosocial behaviour and empathic concern, even surpassing ToM in predictive power (Eggum et al., 2011). Inhibitory control, the ability to self‐regulate impulses and behaviours (Carlson et al., 1998), enables regulation of immediate emotional reactions to focus on others' needs (Decety & Lamm, 2006). It facilitates self‐other distinction around 18 months, allowing suppression of automatic responses for better perspective‐taking (Steinbeis, 2016). A recent meta‐analysis revealed positive correlations between executive functions and empathy in adults (Yan et al., 2020), supporting inhibitory control's role throughout life in modulating empathic responses. MSC is the set of beliefs individuals have about their own prosocial behaviours and moral qualities and is closely related to prosocial behaviours through self‐consistency (Blasi, 1983). Empirical evidence shows a positive, reciprocal relation between MSC and comforting behaviour in pre‐school children (Söldner & Paulus, 2024; Sticker et al., 2021). Temperamental surgency is characterized by traits such as high activity level, impulsivity and high‐intensity pleasure, reflecting a tendency towards exuberance, sociability and positive anticipation (Rothbart et al., 2001). Surgency has been associated with increased comforting behaviours in children (Rothbart et al., 1994). Conversely, studies have observed reduced empathy and comforting behaviours and increased self‐distress in shy children (Eisenberg et al., 2019).

### The current study

The present study has two primary goals. First, we use LPA to statistically derive profiles of children's reactions to others' emotional distress in a sample of 3.5‐ to 6‐year‐old German and Canadian children. Based on Hoffman's (2000) theoretical framework, we expect to identify distinct profiles reflecting individual differences in empathy‐related responses (H1). We anticipate that profiles will vary in their levels of empathic concern, comforting behaviours, self‐distress and avoidance, potentially including: (a) a *prosocial profile* characterized by high empathic concern and active comforting behaviours, reflecting successful transformation of empathic concern into comforting; (b) an *empathic profile* distinguished by high hypothesis testing and empathic concern but limited comforting, suggesting empathic arousal without the social‐cognitive skills to translate concern into effective comforting; (c) a *self‐distressed profile* where children experience egoistic drift, becoming overwhelmed by their own emotions and showing heightened self‐distress that constrains their capacity for empathic concern and comforting; and (d) an *uninvolved profile* characterized by disengagement, minimal empathic concern and low comforting, reflecting avoidance strategies.

We examine how these statistically derived profiles relate to social–cognitive and social–emotional competencies. Based on the link between ToM and prosocial behaviour (Decety & Svetlova, 2012), we expect ToM to be more advanced in profiles characterized by high comforting (prosocial and empathic profiles) compared to profiles with low comforting (self‐distressed and uninvolved profiles; H2). Second, emotion comprehension should distinguish profiles characterized by higher empathic concern from those characterized by self‐distress or disengagement, given its theoretical link to empathic concern (Decety, 2010). We therefore expect the empathic and prosocial profiles to show the highest emotion comprehension (H3). We further expect inhibitory control to be more pronounced in the prosocial profile, as it supports the regulation of empathic arousal necessary for effective helping (Decety & Lamm, 2006; H4). We expect MSC to be higher in the prosocial profile, reflecting the motivation to act self‐consistently with beliefs (Blasi, 1983; H5). Finally, we expect surgency to be lower in the self‐distressed profile (Rothbart et al., 1994), as shy, less exuberant children show increased self‐distress in response to others' emotions, and higher in the prosocial profile, given the link between surgency and comforting behaviours (H6).

## METHOD

This study uses data from the first measurement point of a multi‐site, longitudinal study conducted at two locations. Each child was tested once in an individual session. Data were collected at affiliated universities. Task procedures and coding information are in the Supplemental Material.

### Participants

The final sample comprised 362 German (*n* = 213) and Canadian (*n* = 149) children aged 3 to 6 years without diagnosed developmental or clinical disorders (*M*_age_ = 60.65 months, *SD* = 11.43, range = 42–83 months, 180 girls). 32 additional children were excluded from this study due to missing data in the pain simulation task caused by camera (*n* = 4), procedural (*n* = 10) or experimenter (*n* = 18) errors. The sample size exceeds the recommended minimum of 300 cases for reliable LPA results (Weller et al., 2020) and is larger than that in comparable LPA studies in developmental psychology (Newton et al., 2016; Schachner et al., 2018; Song et al., 2023).

The sample was predominantly White (67.40%), with highly educated parents (first caregivers: *M* = 10.28 years vocational training; second caregivers: *M* = 9.69 years). Twenty families were single‐parent households, and 186 children were bilingual (66.67% from the Canadian sample). Families were recruited through mail invitations, social media, municipal lists, research databases and visits to family events or kindergartens. The study received ethics approval from participating universities. Parents provided consent and received travel allowances; children received small games and certificates.

### Procedure

After warm‐up, children were taken to a testing room where trained experimenters administered tasks in fixed order (pain simulation, ToM scale, Flanker task, emotion comprehension, MSC puppet interview), framed as games. Sessions were videotaped and lasted approximately 90 min. Children completed additional tasks before and in between the tasks of the current study as part of a larger project while parents completed questionnaires. All experimenters wore surgical masks due to COVID‐19 protocols.

### Pain simulation task: Reaction to another person's distress

The pain simulation task assesses empathy‐related responses to emotional distress (Knafo et al., 2008; Young et al., 1999). While children sat across from the experimenter at a table, the experimenter pretended to injure their finger by letting a clipboard snap back with a loud noise, then acted as if in pain.

#### Procedure

The experimenter followed a scripted pain simulation (Bandstra et al., 2011; Zahn‐Waxler et al., 1992) with seven cues at 10‐s intervals, displaying verbal (‘Ouch!’) and non‐verbal expressions (facial distress, rubbing thumb, shaking hand). The sequence ended with verbal indication that pain had subsided. The experimenter maintained eye contact during verbal cues, lowered gaze otherwise and did not respond to children's reactions to ensure consistency.

#### Coding

Children's reactions were coded using scales adapted from previous studies (Bandstra et al., 2011; Knafo et al., 2008; Young et al., 1999; Zahn‐Waxler et al., 1992). *Hypothesis testing* measured attempts to understand distress (0–4 scale: 0 = none, 4 = repeated sophisticated attempts). *Empathic concern* assessed facial, vocal and gestural expressions of concern (0–3 scale: 0 = no concern, 3 = great concern). *Comforting* measured prosocial behaviours including physical/verbal comfort, advice, helping or distracting (0–3 scale: 0 = none, 3 = physical comforting or seeking adult help). *Self‐distress* coded self‐focused negative verbal, facial and postural responses like freezing or self‐soothing (0–3 scale: 0 = none, 3 = crying). *Avoidance* measured disengagement strategies like gaze aversion or ignoring (0–4 scale: 0 = engaged throughout, 4 = complete disengagement). Three coders who were blind to the study hypotheses were trained to ensure consistency across sites. Two primary coders handled their respective German and Canadian sites, with the German coder also coding ~25% of Canadian data for cross‐site reliability. Given language barriers, an additional reliability coder was appointed, who coded ~23% of the German data. Inter‐rater reliability ranged from ICC = .81 to .93 (Table 1). Cases with significant experimenter errors (e.g. procedure shortened by >25% of the duration, omitted cues or script changes altering task meaning) were excluded.

**TABLE 1:** Inter‐rater reliability of dimensions (ICC).

Variable	German sample	Canadian sample
Hypothesis testing	.82 [95% CI: .70, .90]	.90 [95% CI: .81, .95]
Empathic concern	.81 [95% CI: .68, .89]	.90 [95% CI: .82, .95]
Comforting	.87 [95% CI: .78, .93]	.93 [95% CI: .86, .96]
Self‐distress	.83 [95% CI: .71, .90]	.81 [95% CI: .67, .90]
Avoidance	.81 [95% CI: .69, .89]	.88 [95% CI: .78, .93]

*Note*: Reliability samples comprised 48 German children (22.54%) and 38 Canadian children (24.84%).

### Theory of Mind

ToM was measured using a computerized adaptation of Wellman and Liu's (2004) mentalizing scale with five items: Diverse Desires, Diverse Beliefs, Knowledge Access, Contents False Belief and Explicit False Belief. The task discontinued after two successive failures. Children responded at a computer while experimenters logged answers via Qualtrics. Coding validity was confirmed through video accuracy checks. Sum scores ranged from 0 to 5.

### Emotion comprehension

The Test of Emotion Comprehension (TEC; Pons & Harris, 2000) used a picture book with gender‐matched protagonists. Experimenters showed cartoon scenes, narrated stories and asked children to select from four emotions (two negative, two non‐negative) reflecting the protagonist's feelings. The test covered nine components including Recognition of emotions, Understanding the external cause, Desire, Belief, Reminder, Regulation, Hiding, Mixed and Morality. Only physical pointing was accepted; verbal responses required clarification. Experimenters live‐coded on tablets with verification through video codings. Sum scores ranged from 0 to 9, calculated only when all items were completed.

### Inhibitory control

The Flanker task (Zelazo et al., 2013) required children to identify a central fish's direction while flanked by fish pointing the same (congruent) or opposite (incongruent) direction. Trials began with a fixation cross (1000–1500 ms), ‘Middle’ prompt (1000 ms), then five fish appeared with flankers appearing 100 ms before the central target. Children had 10 s to respond via button press. The task included practice (4 trials) and testing phases (24 trials: 16 congruent, 8 incongruent). Data were pre‐processed following Zelazo et al. (2013): Trials with reaction times below 100 ms or exceeding 3 *SD*s from each participant's mean RT were excluded to remove pre‐mature responses and attentional lapses. Inhibitory control was measured as proportion of correct responses on incongruent trials (0–1 scale), with higher scores indicating better performance.

### Moral self‐concept

We adapted Sticker et al.'s (2021) puppet interview using three comforting items. Children viewed gender‐matched puppet pairs expressing opposing preferences about comforting behaviours. After the experimenter wiggled the speaking puppet, children chose which puppet they resembled and whether ‘a little’ or ‘a lot’ like that puppet. Responses were coded on a 5‐point scale from 1 (non‐prosocial) to 5 (prosocial). Items were presented in mixed order with counterbalanced positioning. MSC scores averaged across items ranged 1–5 (Cronbach's *α* = .81).

### Surgency

Surgency was assessed using the CBQ very short form's 12‐item subscale for ages 3–7 (Putnam & Rothbart, 2006). Parents rated their child's reactions over 6 months on a 7‐point scale (1 = extremely untrue to 7 = extremely true). Scores were calculated as means with reverse‐scoring specified items, ranging from 1 (very low) to 7 (very high surgency). An example item: ‘My child often rushes into new situations’. Cronbach's α was .75.

### Statistical analysis

Statistical analyses used R version 4.5.1 (R Core Team, 2025) and RStudio (RStudio Team, 2024) on Mac. To test the first hypothesis, LPA was performed using *tidyLPA* (Rosenberg et al., 2018) to identify distinct subpopulations based on empathic reaction variables. LPA reveals how emotional and behavioural reactions interact across different profiles (Morin & Litalien, 2019; Weller et al., 2020). Profiles were estimated using maximum with conservative constraints fixed indicator variances across profiles and set covariances to 0. *TidyLPA* uses agglomerative hierarchical clustering for initialization. Model selection considered interpretability, AIC, BIC, sample size‐adjusted BIC, entropy, bootstrap likelihood ratio test *p*‐values and theoretical considerations (Christner & Paulus, 2022; Schachner et al., 2018). Follow‐up analyses used modal assignment, assigning participants to their highest‐probability profile. High entropy (.86) suggests minimal uncertainty in profile membership. Fourteen multivariate outliers identified via Mahalanobis distance were retained as profile patterns remained consistent.

Age differences among profiles were evaluated using ANOVA followed by Tukey's HSD post‐hoc tests, gender distribution across profiles was assessed via Chi‐square test. ANCOVAs examined whether ToM, emotion comprehension, inhibitory control, MSC and surgency differed between profiles, controlling for age. Significant results were followed by pairwise comparisons on estimated marginal means. Benjamini–Hochberg correction was applied for pairwise comparisons. This adjustment controls the false discovery rate while maintaining appropriate statistical power for exploratory profile comparisons (Benjamini & Hochberg, 1995), which is suitable for the exploratory nature of LPA follow‐up analyses.

Missing data affected ToM (*n* = 19), TEC (*n* = 31), MSC (*n* = 23), Flanker (*n* = 66) and CBQ (*n* = 15) due to experimenter/computer errors or incomplete responses. Data were not missing completely at random (*p* = .019; Little, 1988). We used predictive mean matching imputation via the *mice* package (van Buuren & Groothuis‐Oudshoorn, 2011). Imputed and complete case analyses showed consistent patterns. Data and analysis scripts will be publicly available after a five‐year embargo period (https://osf.io/fmbx6/). The study was not pre‐registered.

## RESULTS

On average, children displayed moderate‐to‐low levels of hypothesis testing, empathic concern, comforting, self‐distress and avoidance (Table 2; see Data S1 for descriptive statistics and correlations of all variables).

**TABLE 2:** Descriptive statistics and correlations of pain simulation variables.

	*M*	*SD*	Range	(1)	(2)	(3)	(4)	(5)
Hypothesis testing (1)	1.69	1.02	0–4	1				
Empathic concern (2)	1.11	0.96	0–3	.52***	1			
Comforting (3)	0.55	0.75	0–3	.34***	.56***	1		
Self‐distress (4)	0.67	0.65	0–3	−.29***	−.43***	−.41***	1	
Avoidance (5)	0.76	1.12	0–4	−.07	−.27***	−.16**	−.01	1

** *p* < .01.

*** *p* < .001.

### Latent profile analysis

**FIGURE 1:**
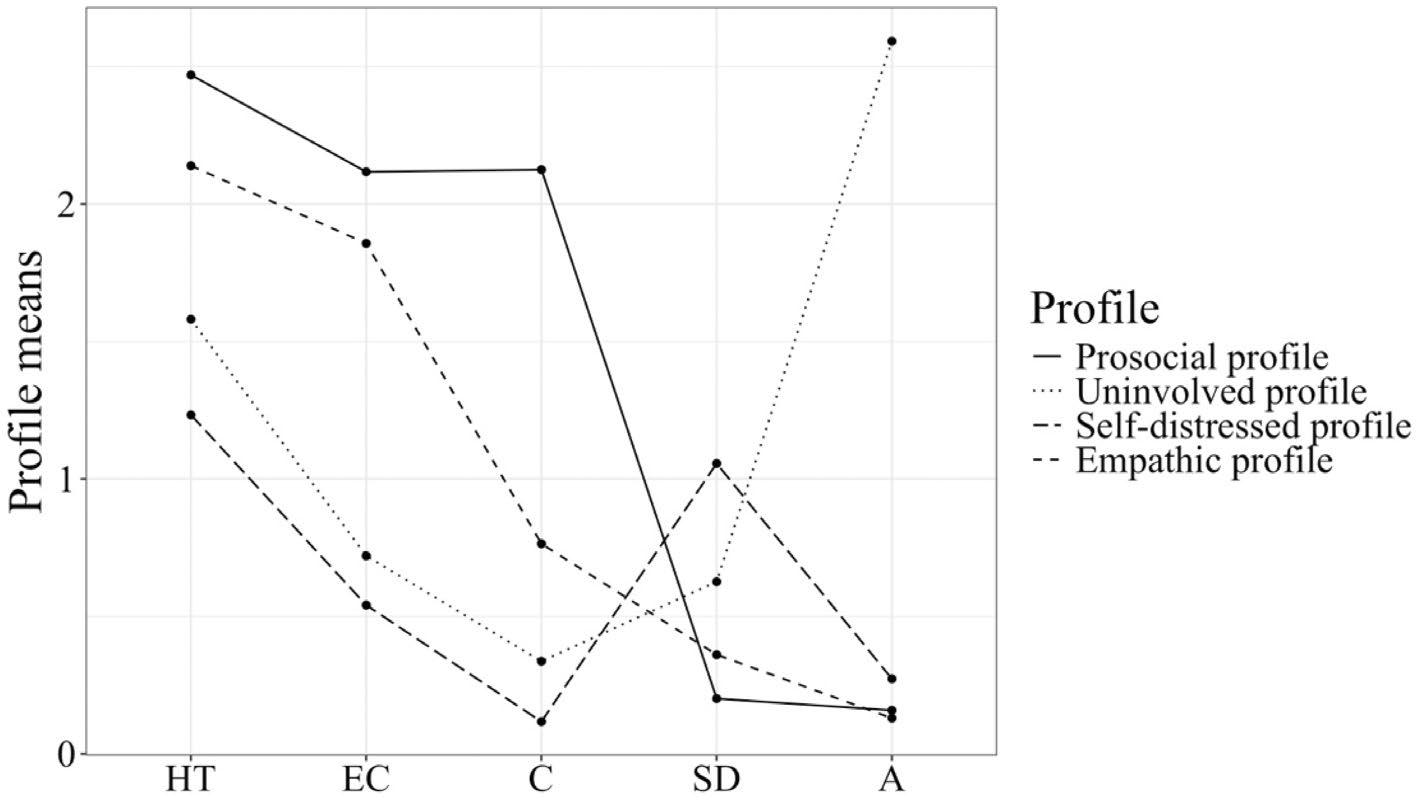
Means of the four latent profiles on the variables hypothesis testing (HT), empathic concern (EC), comforting (C), self‐distress (SD) and avoidance (A).

The four‐profile model was selected as optimal based on fit indices and theoretical considerations (see Table 3). This model showed high entropy (.86) and excellent classification accuracy, with average posterior probabilities ranging from .89 to .99 across all profiles, indicating effective class distinction and minimal classification uncertainty. The model also showed a significant BLRT *p*‐value (*p* = .009), demonstrating meaningful improvement over the three‐profile model. While the six‐profile model had lower AIC/BIC values, the four‐profile model provided the best balance of complexity and interpretability, aligning with theoretical expectations. The four profiles aligned with Hoffman's (2000) framework distinguishing empathic concern and egoistic drift while also capturing an avoidance‐based response, whereas the six‐profile model produced fragmented subgroups without clear theoretical distinction. Figure 1 shows mean scores across all measured variables for the four latent profiles.

**TABLE 3:** Fit indices of LPA for different numbers of profiles.

Number of profiles	AIC	BIC	SABIC	Entropy	BLRT *p*‐value
1	4710.81	4749.73	4718.00	1.00	NA
2	4367.86	4430.13	4379.37	.80	.009
3	4205.49	4291.11	4221.31	.88	.009
4	4168.12	4277.09	4188.25	.86	.009
5	4180.17	4312.49	4204.62	.74	1.00
6	4109.38	4265.04	4138.14	.79	.009

Abbreviations: AIC, Akaike information criterion; BIC, Bayes information criterion; BLRT—bootstrap likelihood ratio test; SABIC, sample‐adjusted BIC.

The first profile (*n* = 37) was characterized by high levels of hypothesis testing, empathic concern and comforting, with low self‐distress and avoidance; we labelled this the ‘prosocial profile’. The second profile (*n* = 86) showed high avoidance, moderate hypothesis testing and self‐distress, and low empathic concern and comforting; we termed this the ‘uninvolved profile’. The third profile (*n* = 138) exhibited high self‐distress, moderate hypothesis testing and low empathic concern, avoidance and comforting; we called this the ‘self‐distressed profile’. The fourth profile (*n* = 101) displayed high hypothesis testing and empathic concern, moderate comforting and low self‐distress and avoidance; we labelled this the ‘empathic profile’. Mean scores for each profile are detailed in Table 4.

**TABLE 4:** Sample size and mean scores of the four latent profiles on the pain simulation task variables.

Class	% of sample	HT	EC	C	*SD*	A	Name
1	10.22	2.43	2.01	2.16	0.22	0.16	Prosocial profile
2	23.76	1.58	0.72	0.34	0.63	2.59	Uninvolved profile
3	38.12	1.19	0.52	0.09	1.06	0.27	Self‐distressed profile
4	27.90	2.21	1.89	0.76	0.36	0.13	Empathic profile

*Note*: Hypothesis testing (HT; range 0–4), empathic concern (EC; range 0–3), comforting (C; range 0–3), self‐distress (*SD*; range 0–3) and avoidance (A; range 0–4).

Post‐hoc analyses revealed significant differences between profiles across all variables. The ‘prosocial’ and ‘empathic profiles’ showed similar hypothesis testing and empathic concern (*p* > .050), but differed in comforting, with the prosocial profile showing more comforting behaviours (*p* < .001). The ‘self‐distressed profile’ showed the lowest hypothesis testing, empathic concern and comforting, plus the highest self‐distress (all *p* < .001). The ‘uninvolved profile’ showed the highest avoidance and low levels of empathic concern and comforting. Detailed comparisons are in supplemental material.

### Differences between profiles (Figure 2)

**FIGURE 2:**
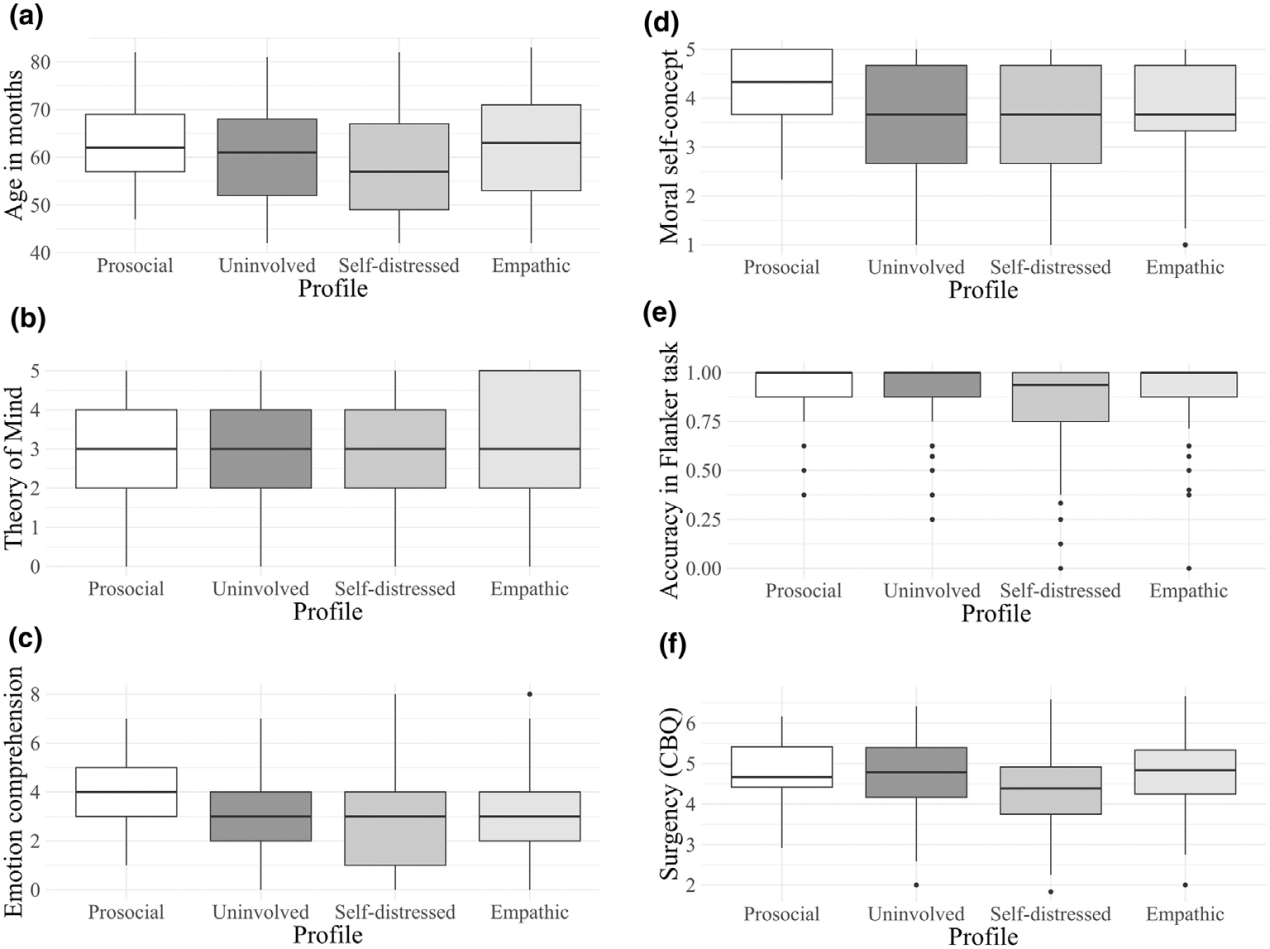
Boxplots of variables across distinct profiles. (a) age in months; (b) Theory of Mind; (c) emotion comprehension; (d) moral self‐concept; (e) Flanker task; (f) surgency.

The ANOVA revealed significant age differences across profiles (*F*(3, 358) = 3.55, *p* = .015, *η* ^*2*^ = 0.03). Children in the self‐distressed profile were significantly younger than those in the prosocial profile (*p* = .046). Gender distribution did not differ significantly across profiles (χ^2^(3) = 1.95, *p* = .583, Cramér's *V* = 0.07). Therefore, age was included as a covariate in all subsequent analyses, while gender was not. Adjusted means and 95% confidence intervals for all variables, controlling for age, are presented in Table 5.

**TABLE 5:** Adjusted means and 95% confidence intervals for variables by profile, controlling for age.

	Prosocial	Uninvolved	Self‐distressed	Empathic
Theory of Mind	3.08 [2.65, 3.52]	3.2 [2.92, 3.48]	3.21 [2.99, 3.43]	3.18 [2.92, 3.45]
Emotion comprehension*	3.66 [3.18, 4.15]	3.03 [2.72, 3.35]	3.14 [2.89, 3.39]	3.07 [2.77, 3.36]
Moral self‐concept*	4.14 [3.78, 4.5]^a^	3.55 [3.32, 3.79]^b^	3.64 [3.46, 3.83]^ab^	3.71 [3.49, 3.93]^ab^
Inhibitory control	0.89 [0.83, 0.95]	0.88 [0.84, 0.92]	0.86 [0.83, 0.89]	0.88 [0.85, 0.92]
Surgency***	4.79 [4.51, 5.06]^a^	4.78 [4.6, 4.95]^a^	4.35 [4.21, 4.49]^b^	4.74 [4.58, 4.91]^a^

*Note*: Values are estimated marginal means with 95% confidence intervals. Within each row, values with different superscripts differ significantly (Benjamini–Hochberg–adjusted *p* < .050). *p < .05, ***p < .001 for overall ANCOVA effect.

#### Theory of Mind

The ANCOVA showed no significant differences in ToM scores across profiles when controlling for age (*F*(3, 357) = 0.35, *p* = .792, partial *η* ^*2*^ = 0.001).

#### Emotion comprehension

Results indicated significant overall differences in emotion comprehension across profiles when controlling for age (*F*(3, 362) = 4.55, *p* = .004, partial *η* ^*2*^ = 0.01). However, Benjamini–Hochberg–adjusted pairwise comparisons did not reach significance.

#### Moral self‐concept

The ANCOVA revealed significant differences in MSC across profiles when controlling for age (*F*(3, 357) = 3.29, *p* = .021, partial *η* ^*2*^ = 0.02). Benjamini–Hochberg–adjusted pairwise comparisons indicated that children in the prosocial profile had higher MSC than those in the uninvolved profile (*p* = .047).

#### Inhibitory control

Profiles did not differ significantly in Flanker accuracy scores when controlling for age (*F*(3, 357) = 1.79, *p* = .149, partial *η* ^*2*^ = 0.003).

#### Surgency

Significant differences in surgency emerged across profiles when controlling for age (*F*(3, 357) = 7.02, *p* < .001, partial *η* ^*2*^ = 0.05). Children in the self‐distressed profile showed significantly lower surgency than those in prosocial (*p* = .011), uninvolved (*p* = .001) and empathic (*p* = .001) profiles.

#### Research site

To exploratively examine whether profile membership differed by research site (Canada vs. Germany), we conducted a multinomial logistic regression with profile as the outcome and site and age as predictors. German children were significantly more likely than Canadian children to be classified into the uninvolved relative to the empathic profile (OR = 1.82, *p* = .041). All other contrasts were non‐significant (*p*s > .210).

## DISCUSSION

Understanding why some children comfort others in distress while other children become overwhelmed or disengage is central to understanding empathy and prosocial behaviour in early childhood (Dunfield, 2014; Spinrad & Eisenberg, 2019). This study statistically derived profiles of empathy‐related behaviours in children aged 3.5 to 6 years through a person‐centred approach. LPA identified four distinct profiles—prosocial, uninvolved, self‐distressed and empathic—complementing existing variable‐centred research by demonstrating how empathy‐related responses co‐occur within individuals. These profiles align with Hoffman's (2000) distinction between empathic concern (prosocial and empathic profiles) and egoistic drift (self‐distressed profile), while the uninvolved profile represents an avoidance‐based response. The distinction between prosocial and empathic profiles reveals that empathic concern does not automatically translate into comforting behaviours, even in early childhood.

### Empathy‐related behavioural profiles

The four profiles provide empirical evidence for theoretical distinctions in how children process and respond to others' distress. Hoffman's (2000) theory proposes that empathy can follow two pathways depending on cognitive and regulatory capacities. Self‐other differentiation enables children to transform empathic distress into empathic concern, a prosocial motivation to comfort (Batson et al., 1986; Paulus, 2018). However, when distress cues are highly salient and exceed regulatory capacities, empathic over‐arousal occurs, leading to egoistic drift: The observer becomes pre‐occupied with personal distress and turns attention away from the victim.

The *prosocial* and *empathic profiles* exemplify successful transformation of empathic distress into empathic concern, maintaining other‐oriented focus with low self‐distress. Both show comparably high empathic concern and hypothesis testing yet differ markedly in comforting behaviours, demonstrating that empathic concern is necessary but insufficient for comforting (Eisenberg et al., 2015). The *self‐distressed profile* exemplifies egoistic drift. These children show elevated self‐distress coupled with significantly lower empathic concern and almost no comforting behaviours. This pattern reflects empathic over‐arousal overwhelming regulatory capacity, shifting attention from the victim's needs to the child's own emotional state. The *uninvolved profile* reveals a mechanism not explicitly addressed in Hoffman's framework: Rather than experiencing empathic over‐arousal, these children show high avoidance with only moderate self‐distress. This pattern suggests active disengagement that may prevent initial empathic arousal from occurring, representing a fundamentally different barrier to comforting than egoistic drift (Stifter & Braungart, 1995).

Person‐centred approaches reveal that profiles are characterized by distinct configurations reflecting the presence and absence of certain behavioural reactions, where near‐zero means for certain behaviours reflect meaningful response patterns rather than measurement limitations (e.g. Song et al., 2023). The uninvolved profile shows consistently low levels across all behaviours, while the prosocial profile exhibits high comforting with minimal self‐distress. These contrasting patterns indicate that children employ qualitatively different response strategies when confronted with others' distress (Hoffman, 2000).

### Differences between profiles

Temperamental surgency and MSC distinguished profiles. Children in the *self‐distressed profile* showed lower surgency than all other profiles, while children in the *prosocial profile* had higher MSC than those in the *uninvolved profile*. Additionally, overall effects suggested a role for emotion comprehension in profile membership, although Benjamini–Hochberg–adjusted pairwise comparisons did not reach significance for this variable. ToM and inhibitory control did not differ significantly across profiles. These patterns suggest that temperamental and motivational factors distinguish empathy‐related response profiles more reliably than social‐cognitive abilities in pre‐schoolers.

Children in the *self‐distressed profile* showed significantly lower surgency compared with all other profiles, consistent with theoretical accounts linking temperament to other‐oriented behaviours (Hampson, 1981). Children with lower surgency tend to display behavioural wariness and withdrawal in social situations, particularly with unfamiliar peers (Dollar & Stifter, 2012), and demonstrate lower use of adaptive emotion regulation strategies (Planalp & Braungart‐Rieker, 2015). Importantly, the *self‐distressed profile* exhibited comparable ToM and emotion comprehension to other profiles despite lower comforting behaviours. This pattern suggests that their reduced comforting reflects performance deficits rather than competence deficits, that is they possess the social–cognitive understanding necessary for comforting but fail to express it behaviourally, consistent with accounts proposing that social fear and evaluative concerns suppress empathy‐related behaviours in unfamiliar situations (Coplan et al., 2020). Notably, the *uninvolved profile* exhibited surgency levels comparable with the prosocial and empathic profiles, despite similarly low empathic concern and comforting as the self‐distressed profile. This indicates that the *uninvolved profile*'s lack of comforting behaviours stems from different mechanisms, such as active disengagement that prevents empathic arousal from occurring, rather than temperamental constraint. Surgency did not distinguish the *prosocial profile* from the *empathic* and *uninvolved profiles*, suggesting that high surgency is neither a necessary nor sufficient characteristic of profiles marked by high comforting behaviours in pre‐schoolers.

The significant difference in MSC between the prosocial and uninvolved profiles provides suggestive evidence for the role of moral self‐representations in empathy‐related behaviours. Children in *the prosocial profile* exhibited stronger MSC than those in the uninvolved profile, aligning with theoretical frameworks proposing that moral self‐representations facilitate prosocial action by motivating to act consistently with one's self‐concept (Blasi, 1983). However, the similarity between prosocial and empathic profiles in emotion comprehension and MSC was unexpected, given their marked differences in comforting behaviours. Previous research with pre‐schoolers shows that stronger MSC relates to more comforting behaviours (Söldner & Paulus, 2024; Sticker et al., 2021). The empathic profile's comparable MSC despite lower comforting suggests that MSC alone is insufficient to guarantee prosocial action during early childhood, when MSC is still developing coherence and behavioural consistency (Söldner & Paulus, 2024). Descriptively, the empathic profile showed slightly higher self‐distress than the prosocial profile, suggesting that emotion regulation difficulties could hinder translating empathic concern and MSC into comforting behaviours.

Profile comparisons revealed age differences, with children in the *prosocial profile* being older than those in other profiles, particularly the self‐distressed profile. This aligns with developmental theories proposing that children's empathic concern and ability to comfort others develop with emotional and social skills (Hoffman, 2000). However, profile differences in surgency remained significant when controlling for age, indicating that behavioural patterns reflect individual differences beyond developmental changes.

We anticipated that ToM would distinguish profiles based on theoretical frameworks emphasizing cognitive perspective‐taking in empathy development (Decety & Svetlova, 2012). However, ToM did not differ across profiles, challenging the notion that individual differences in empathy during early childhood are primarily driven by cognitive capabilities (Imuta et al., 2016). This may reflect developmental timing. By age 3, most cognitive pre‐requisites for empathic concern and comforting, including basic perspective‐taking abilities, may be sufficiently developed across children (Hoffman, 2000), reducing their discriminatory power. Alternatively, empathy‐related behavioural patterns may be driven more by affective and motivational processes than cognitive capacities, with effective comforting depending more on prior experience and familiarity with social scripts than sophisticated perspective‐taking (Eisenberg et al., 1996).

Similarly, inhibitory control did not significantly differ across profiles, contrary to our expectations and theoretical accounts linking self‐regulation to prosocial behaviour (Decety & Lamm, 2006). This suggests a more complex relation between cognitive control and empathy‐related behaviours than anticipated. One possibility is that emotion regulation—involving broader skills for managing emotional experiences and expressions—may be more relevant for distinguishing empathy‐related behavioural patterns than the specific cognitive control processes measured by our inhibitory control task (Fabes et al., 1994; Yavuz et al., 2024). Alternatively, methodological constraints may have limited our ability to detect differences. The Flanker task was designed to produce robust experimental effects by minimizing variability between participants, but this makes it poorly suited for reliably distinguishing between individuals (Hedge et al., 2018). The absence of profile differences in inhibitory control may thus reflect measurement limitations rather than absence of differences in cognitive control.

### Limitations and future research

A notable strength of this study is the use of LPA to identify distinct empathy‐related response patterns. However, several limitations must be acknowledged. First, the cross‐sectional design limits causal inferences about whether these profiles reflect stable characteristics or situational responses. Second, our sample size, while adequate for LPA, may have limited power to detect small pairwise differences after correction for multiple comparisons. Third, this study was conducted in a controlled laboratory environment with an adult experimenter, which may not reflect natural behaviours with familiar peers. While children had completed familiarization and other prosocial tasks before the pain simulation, the unfamiliar setting may have amplified temperamental effects, particularly for children low in surgency, as inhibited temperament negatively relates to empathy towards unfamiliar adults (Young et al., 1999). Fourth, this study was conducted shortly after the COVID‐19 pandemic when face masks were required, potentially limiting children's access to facial expression cues. Finally, the sample represents a WEIRD population, limiting generalizability.

Future research should address several directions. Longitudinal designs (e.g. latent transition analysis) would establish whether profiles reflect stable characteristics or developmental trajectories. Examining behavioural responses across distinct contexts, particularly familiar versus unfamiliar partners and laboratory versus naturalistic settings, would clarify whether patterns remain stable or reflect context‐dependent responses.

The exploratory result indicating that German children were more likely to be classified into the uninvolved relative to the empathic profile was unexpected, given that both samples draw from WEIRD populations with broadly comparable cultural contexts. Future research should include explicit measures of cultural socialization practices, such as parental expressivity norms or empathy‐related socialization goals, to identify what drives cross‐national differences in empathy‐related response profiles.

Additionally, investigating how emotion self‐awareness (i.e. recognizing and understanding one's own emotions) and emotion comprehension jointly contribute to empathy‐related response profiles would provide a more comprehensive understanding, as self‐awareness of one's own emotions enhances empathic concern by providing the basis for identifying with others' feelings (Decety & Jackson, 2004).

## CONCLUSION

This study is unique in examining empathy‐related behavioural profiles using a person‐centred approach in pre‐school‐aged children. The four identified profiles reveal that children exhibit qualitatively different patterns of responding to others' emotional distress, with temperamental surgency and MSC emerging as distinguishing factors. These results show that individual differences in empathy‐related responses reflect distinct within‐person configurations of cognitive, affective and temperamental characteristics rather than simply varying levels of a single empathic capacity.

## AUTHOR CONTRIBUTIONS

**Johannes Bullinger:** Conceptualization; investigation; methodology; software; data curation; formal analysis; visualization; writing – original draft. **Natalie Christner:** Conceptualization; methodology; writing – review and editing; supervision. **Radu Urian:** Conceptualization; investigation; methodology; writing – review and editing; validation; software; formal analysis; data curation. **Christina M. Kellermann:** Investigation; data curation; validation; methodology; formal analysis; writing – review and editing; software. **Seleste Beaulieu:** Investigation; data curation; writing – review and editing; methodology. **Nikolaus Steinbeis:** Funding acquisition; project administration; writing – review and editing; methodology; resources. **Kristen A. Dunfield:** Resources; project administration; writing – review and editing; supervision; methodology; funding acquisition. **Markus Paulus:** Methodology; supervision; funding acquisition; writing – review and editing; resources; project administration.

## CONFLICT OF INTEREST STATEMENT

The authors declare no conflicts of interest.

## Supporting information


Data S1

## Data Availability

Data and analysis scripts will be publicly available after a five‐year embargo period (https://osf.io/fmbx6/). The materials necessary to attempt to replicate the findings presented here are publicly accessible in the supplemental material. The analyses presented here were not pre‐registered.

## References

[bjdp70042-bib-0001] Bandstra, N. F. , Chambers, C. T. , McGrath, P. J. , & Moore, C. (2011). The behavioural expression of empathy to others' pain versus others' sadness in young children. Pain, 152(5), 1074–1082. 10.1016/j.pain.2011.01.024 21349641

[bjdp70042-bib-0002] Batson, C. D. , Bolen, M. H. , Cross, J. A. , & Neuringer‐Benefiel, H. E. (1986). Where is the altruism in the altruistic personality? Journal of Personality and Social Psychology, 50(1), 212–220. 10.1037/0022-3514.50.1.212

[bjdp70042-bib-0003] Benjamini, Y. , & Hochberg, Y. (1995). Controlling the false discovery rate: A practical and powerful approach to multiple testing. Journal of the Royal Statistical Society, 57, 289–300.

[bjdp70042-bib-0004] Blasi, A. (1983). Moral cognition and moral action: A theoretical perspective. Developmental Review, 3(2), 178–210. 10.1016/0273-2297(83)90029-1

[bjdp70042-bib-0005] Carlson, S. M. , Moses, L. J. , & Hix, H. R. (1998). The role of inhibitory processes in young children's difficulties with deception and false belief. Child Development, 69(3), 672–691. 10.1111/j.1467-8624.1998.tb06236.x 9680679

[bjdp70042-bib-0006] Christner, N. , & Paulus, M. (2022). Varieties of normative understanding and their relation to sharing behavior in preschool children. Journal of Experimental Child Psychology, 224, 105498. 10.1016/j.jecp.2022.105498 35842944

[bjdp70042-bib-0007] Coplan, R. J. , Baldwin, D. , & Wood, K. R. (2020). Shy but getting by: Protective factors in the links between childhood shyness and socio‐emotional functioning. In L. A. Schmidt & K. L. Poole (Eds.), Adaptive shyness (pp. 63–90). Springer. 10.1007/978-3-030-38877-5_4

[bjdp70042-bib-0008] Davidov, M. , Roth‐Hanania, R. , Paz, Y. , Orlitsky, T. , Uzefovsky, F. , & Zahn‐Waxler, C. (2025). Empathy development from birth to three: Advances in knowledge from 2000 to 2025. Infant Behavior and Development, 81, 102144. 10.1016/j.infbeh.2025.102144 40961882

[bjdp70042-bib-0009] Decety, J. (2010). The neurodevelopment of empathy in humans. Developmental Neuroscience, 32(4), 257–267. 10.1159/000317771 20805682 PMC3021497

[bjdp70042-bib-0010] Decety, J. , & Jackson, P. L. (2004). The functional architecture of human empathy. Behavioral and Cognitive Neuroscience Reviews, 3(2), 71–100. 10.1177/1534582304267187 15537986

[bjdp70042-bib-0011] Decety, J. , & Lamm, C. (2006). Human empathy through the lens of social neuroscience. The Scientific World Journal, 6, 1146–1163. 10.1100/tsw.2006.221 16998603 PMC5917291

[bjdp70042-bib-0012] Decety, J. , & Svetlova, M. (2012). Putting together phylogenetic and ontogenetic perspectives on empathy. Developmental Cognitive Neuroscience, 2(1), 1–24. 10.1016/j.dcn.2011.05.003 22682726 PMC6987713

[bjdp70042-bib-0013] Dollar, J. M. , & Stifter, C. A. (2012). Temperamental surgency and emotion regulation as predictors of childhood social competence. Journal of Experimental Child Psychology, 112(2), 178–194. 10.1016/j.jecp.2012.02.004 22414737 PMC4329969

[bjdp70042-bib-0014] Dunfield, K. A. (2014). A construct divided: Prosocial behavior as helping, sharing, and comforting subtypes. Frontiers in Psychology, 5, 1–13. 10.3389/fpsyg.2014.00958 25228893 PMC4151454

[bjdp70042-bib-0015] Dunfield, K. A. , & Kuhlmeier, V. A. (2013). Classifying prosocial behavior: Children's responses to instrumental need, emotional distress, and material desire. Child Development, 84(5), 1766–1776. 10.1111/cdev.12075 23461793

[bjdp70042-bib-0016] Eggum, N. D. , Eisenberg, N. , Kao, K. , Spinrad, T. L. , Bolnick, R. , Hofer, C. , Kupfer, A. S. , & Fabricius, W. V. (2011). Emotion understanding, theory of mind, and prosocial orientation: Relations over time in early childhood. Journal of Positive Psychology, 6(1), 1–18. 10.1080/17439760.2010.536776 PMC3328349 22518196

[bjdp70042-bib-0017] Eisenberg, N. , Betkowski, J. , & Spinrad, T. L. (2013). Age‐related changes in empathy‐related responding. In D. Hermans , B. Rimé , & B. Mesquita (Eds.), Changing Emotions. Psychology Press. http://ebookcentral.proquest.com/lib/nyulibrary‐ebooks/detail.action?docID=1128274

[bjdp70042-bib-0018] Eisenberg, N. , & Eggum, N. D. (2009). Empathic responding: Sympathy and personal distress. In J. Decety & W. Ickes (Eds.), The social neuroscience of empathy (pp. 71–84). The MIT Press. 10.7551/mitpress/9780262012973.003.0007

[bjdp70042-bib-0019] Eisenberg, N. , Fabes, R. A. , & Murphy, B. C. (1996). Parents' reactions to children's negative emotions: Relations to children's social competence and comforting behavior. Child Development, 67(5), 2227–2247. 10.2307/1131620 9022240

[bjdp70042-bib-0020] Eisenberg, N. , Shea, C. L. , Carlo, G. , & Knight, G. P. (1991). Empathy‐related responding and cognition: A “chicken and the egg” dilemma. In W. M. Kurtines & J. L. Gewirtz (Eds.), Handbook of moral behavior and development. Volume 2: Research (pp. 63–88). Lawrence Erlbaum Associates, Inc.

[bjdp70042-bib-0021] Eisenberg, N. , Spinrad, T. L. , & Knafo‐Noam, A. (2015). Prosocial development. In R. M. Lerner (Ed.), Handbook of child psychology and developmental science (7th ed., pp. 1–47). John Wiley & Sons, Inc. 10.1002/9781118963418.childpsy315

[bjdp70042-bib-0022] Eisenberg, N. , Spinrad, T. L. , Taylor, Z. E. , & Liew, J. (2019). Relations of inhibition and emotion‐related parenting to young children's prosocial and vicariously induced distress behavior. Child Development, 90(3), 846–858. 10.1111/cdev.12934 28857139

[bjdp70042-bib-0023] Fabes, R. A. , Eisenberg, N. , Karbon, M. , Troyer, D. , & Switzer, G. (1994). The relations of children's emotion regulation to their vicarious emotional responses and comforting behaviors. Child Development, 65(6), 1678–1693. 10.1111/j.1467-8624.1994.tb00842.x 7859549

[bjdp70042-bib-0024] Gibhardt, S. , Aitken, J. , Low, R. , & Henderson, A. M. E. (2024). Task demands matter in shaping how preschoolers express instrumental helping, comforting, and sharing: A longitudinal analysis. Journal of Experimental Child Psychology, 243, 105929. 10.1016/j.jecp.2024.105929 38663123

[bjdp70042-bib-0025] Hampson, R. B. (1981). Helping behavior in children: Addressing the interaction of a person‐situation model. Developmental Review, 1(2), 93–112. 10.1016/0273-2297(81)90011-3

[bjdp70042-bib-0026] Hedge, C. , Powell, G. , & Sumner, P. (2018). The reliability paradox: Why robust cognitive tasks do not produce reliable individual differences. Behavior Research Methods, 50(3), 1166–1186. 10.3758/s13428-017-0935-1 28726177 PMC5990556

[bjdp70042-bib-0027] Hoffman, M. L. (2000). Empathy and moral development: Implications for caring and justice. Cambridge University Press. 10.1017/CBO9780511805851

[bjdp70042-bib-0028] Imuta, K. , Henry, J. D. , Slaughter, V. , Selcuk, B. , & Ruffman, T. (2016). Theory of mind and prosocial behavior in childhood: A meta‐analytic review. Developmental Psychology, 52(8), 1192–1205. 10.1037/dev0000140 27337508

[bjdp70042-bib-0029] Kienbaum, J. (2014). The development of sympathy from 5 to 7 years: Increase, decline, or stability? A longitudinal study. Frontiers in Psychology, 5, 1–10. 10.3389/fpsyg.2014.00468 24904484 PMC4033067

[bjdp70042-bib-0030] Knafo, A. , Steinberg, T. , & Goldner, I. (2011). Children's low affective perspective‐taking ability is associated with low self‐initiated pro‐sociality. Emotion, 11(1), 194–198. 10.1037/a0021240 21401240

[bjdp70042-bib-0031] Knafo, A. , Zahn‐Waxler, C. , Davidov, M. , Van Hulle, C. , Robinson, J. A. L. , & Rhee, S. H. (2009). Empathy in early childhood: Genetic, environmental, and affective contributions. Annals of the new York Academy of Sciences, 1167, 103–114. 10.1111/j.1749-6632.2009.04540.x 19580557

[bjdp70042-bib-0032] Knafo, A. , Zahn‐Waxler, C. , Van Hulle, C. , Robinson, J. A. L. , & Rhee, S. H. (2008). The developmental origins of a disposition toward empathy: Genetic and environmental contributions. Emotion, 8(6), 737–752. 10.1037/a0014179 19102585

[bjdp70042-bib-0033] Little, R. J. A. (1988). A test of missing completely at random for multivariate data with missing values. Journal of the American Statistical Association, 83(404), 1198–1202. 10.1080/01621459.1988.10478722

[bjdp70042-bib-0034] Malti, T. , Eisenberg, N. , Kim, H. , & Buchmann, M. (2013). Developmental trajectories of sympathy, moral emotion attributions, and moral reasoning: The role of parental support. Social Development, 22(4), 773–793. 10.1111/sode.12031

[bjdp70042-bib-0035] Morin, A. J. S. , & Litalien, D. (2019). Mixture modeling for lifespan developmental research. In Oxford research encyclopedia of psychology. Oxford University Press. 10.1093/acrefore/9780190236557.013.364

[bjdp70042-bib-0036] Newton, E. K. , Thompson, R. A. , & Goodman, M. (2016). Individual differences in toddlers' prosociality: Experiences in early relationships explain variability in prosocial behavior. Child Development, 87(6), 1715–1726. 10.1111/cdev.12631 28262933

[bjdp70042-bib-0037] Paulus, M. (2018). The multidimensional nature of early prosocial behavior: A motivational perspective. Current Opinion in Psychology, 20, 111–116. 10.1016/j.copsyc.2017.09.003 28988024

[bjdp70042-bib-0038] Paulus, M. , Becher, T. , Christner, N. , Kammermeier, M. , Gniewosz, B. , & Pletti, C. (2024). When do children begin to care for others? The ontogenetic growth of empathic concern across the first two years of life. Cognitive Development, 70, 101439. 10.1016/j.cogdev.2024.101439

[bjdp70042-bib-0039] Planalp, E. M. , & Braungart‐Rieker, J. M. (2015). Trajectories of regulatory behaviors in early infancy: Determinants of infant self‐distraction and self‐comforting. Infancy, 20(2), 129–159. 10.1111/infa.12068 25685094 PMC4326065

[bjdp70042-bib-0040] Pons, F. , & Harris, P. L. (2000). Test of emotion comprehension: TEC. Oxford University Press.

[bjdp70042-bib-0041] Putnam, S. P. , & Rothbart, M. K. (2006). Development of short and very short forms of the Children's behavior questionnaire. Journal of Personality Assessment, 87(1), 102–112. 10.1207/s15327752jpa8701_09 16856791

[bjdp70042-bib-0042] R Core Team . (2025). R: A language and environment for statistical computing. R Foundation for Statistical Computing. https://www.R‐project.org/

[bjdp70042-bib-0043] Rosenberg, J. , Beymer, P. , Anderson, D. , van Lissa, C. J. , & Schmidt, J. (2018). tidyLPA: An R package to easily carry out latent profile analysis (LPA) using open‐source or commercial software. The Journal of Open Source Software, 3(30), 978. 10.21105/joss.00978

[bjdp70042-bib-0044] Rothbart, M. Κ. , Ahadi, S. A. , & Hershey, K. L. (1994). Temperament and social behavior in childhood. Merrill‐Palmer Quarterly, 40(1), 21–39. http://www.jstor.org/stable/23087906

[bjdp70042-bib-0045] Rothbart, M. Κ. , Ahadi, S. A. , Hershey, K. L. , & Fisher, P. (2001). Investigations of temperament at three to seven years: The Children's behavior questionnaire. Child Development, 72(5), 1394–1408. 10.1111/1467-8624.00355 11699677

[bjdp70042-bib-0046] Roth‐Hanania, R. , Davidov, M. , & Zahn‐Waxler, C. (2011). Empathy development from 8 to 16 months: Early signs of concern for others. Infant Behavior and Development, 34(3), 447–458. 10.1016/j.infbeh.2011.04.007 21600660

[bjdp70042-bib-0047] RStudio Team . (2024). RStudio: Integrated development environment for R. Posit Software, PBC. http://www.rstudio.com/

[bjdp70042-bib-0048] Schachner, A. C. W. , Newton, E. K. , Thompson, R. A. , & Goodman‐Wilson, M. (2018). Becoming prosocial: The consistency of individual differences in early prosocial behavior. Early Childhood Research Quarterly, 43, 42–51. 10.1016/j.ecresq.2018.01.001

[bjdp70042-bib-0049] Shoshani, A. (2024). The roots of compassion in early childhood: Relationships between theory of mind and attachment representations with empathic concern and prosocial behavior. Journal of Experimental Child Psychology, 242, 105880. 10.1016/j.jecp.2024.105880 38368743

[bjdp70042-bib-0050] Söldner, L. , & Paulus, M. (2024). I help, therefore, I am? – A registered report on longitudinal inter‐relations of the three‐dimensional moral self‐concept and prosocial behaviours in preschool children. British Journal of Developmental Psychology, 42(2), 257–284. 10.1111/bjdp.12481 38483075

[bjdp70042-bib-0051] Song, Y. , Broekhuizen, M. , & Dubas, J. S. (2023). Prosocial development throughout the toddlerhood: A person‐centred approach. Infant and Child Development, 32(6), 1–17. 10.1002/icd.2457

[bjdp70042-bib-0052] Spinrad, T. L. , & Eisenberg, N. (2019). Prosocial emotions. In V. LoBue , K. Pérez‐Edgar , & K. A. Buss (Eds.), Handbook of emotional development (pp. 351–372). Springer International Publishing. 10.1007/978-3-030-17332-6_14

[bjdp70042-bib-0053] Steinbeis, N. (2016). The role of self‐other distinction in understanding others' mental and emotional states: Neurocognitive mechanisms in children and adults. Philosophical Transactions of the Royal Society, B: Biological Sciences, 371(1686), 20150074. 10.1098/rstb.2015.0074 PMC4685520 26644593

[bjdp70042-bib-0054] Sticker, R. M. , Christner, N. , Pletti, C. , & Paulus, M. (2021). The moral self‐concept in preschool children: Its dimensions and relation to prosocial behaviors. Cognitive Development, 58, 101033. 10.1016/j.cogdev.2021.101033

[bjdp70042-bib-0055] Stifter, C. A. , & Braungart, J. M. (1995). The regulation of negative reactivity in infancy: Function and development. Developmental Psychology, 31(3), 448–455. 10.1037/0012-1649.31.3.448

[bjdp70042-bib-0056] van Buuren, S. , & Groothuis‐Oudshoorn, K. (2011). MICE: Multivariate imputation by chained equations in R. Journal of Statistical Software, 45(3), 1–67. 10.18637/jss.v045.i03

[bjdp70042-bib-0057] Weller, B. E. , Bowen, N. K. , & Faubert, S. J. (2020). Latent class analysis: A guide to best practice. Journal of Black Psychology, 46(4), 287–311. 10.1177/0095798420930932

[bjdp70042-bib-0058] Wellman, H. M. , & Liu, D. (2004). Scaling of theory‐of‐mind tasks. Child Development, 75(2), 523–541. 10.1111/j.1467-8624.2004.00691.x 15056204

[bjdp70042-bib-0059] Williams, A. , O'Driscoll, K. , & Moore, C. (2014). The influence of empathic concern on prosocial behavior in children. Frontiers in Psychology, 5, 425. 10.3389/fpsyg.2014.00425 24860537 PMC4026684

[bjdp70042-bib-0060] Yan, Z. , Hong, S. , Liu, F. , & Su, Y. (2020). A meta‐analysis of the relationship between empathy and executive function. PsyCh Journal, 9(1), 34–43. 10.1002/pchj.311 31394592

[bjdp70042-bib-0061] Yavuz, H. M. , Colasante, T. , Galarneau, E. , & Malti, T. (2024). Empathy, sympathy, and emotion regulation : A meta‐analytic review. Psychological Bulletin, 150(1), 27–44. 10.1037/bul0000426 38376910

[bjdp70042-bib-0062] Young, S. K. , Fox, N. A. , & Zahn‐Waxler, C. (1999). The relations between temperament and empathy in 2‐year‐olds. Developmental Psychology, 35(5), 1189–1197. 10.1037//0012-1649.35.5.1189 10493645

[bjdp70042-bib-0063] Zahn‐Waxler, C. , Radke‐Yarrow, M. , Wagner, E. , & Chapman, M. (1992). Development of concern for others. Developmental Psychology, 28(1), 126–136. 10.1037/0012-1649.28.1.126

[bjdp70042-bib-0064] Zelazo, P. D. , Anderson, J. E. , Richler, J. , Wallner‐Allen, K. , Beaumont, J. L. , & Weintraub, S. (2013). NIH toolbox cognition battery (CB): Measuring executive function and attention. Monographs of the Society for Research in Child Development, 78(4), 16–33. 23952200 10.1111/mono.12032

