# Supplementary material for: Individual differences in empathy‐related responses in early childhood: A person‐centred approach
Source: Br J Dev Psychol. 2026 Mar 26;44(3):671–88. doi: 10.1111/bjdp.70042 (PMC13452005; doi:10.1111/bjdp.70042)
Supplement: Supplementary file 1 [file BJDP-44-671-s001.docx]

**Supplemental material for “Individual Differences in Empathy-Related Responses in Early Childhood: A Person-Centered Approach”**

The supplemental material includes the full task script of pain simulation task (A), the coding scheme of the pain simulation task (B), the full task script of the puppet interview to assess the moral self-concept (C), and the descriptive results (D).

**A) Procedure of pain simulation task**

| **Time in seconds** | **Instructions** | **Facial Expression** |
| --- | --- | --- |
| “Now I have to look again briefly in my sheets to see what we’re going to play next.”  E is putting away the materials from the previous task and looking through his/her documents. S/he gets his/her **left thumb** caught by the **clipboard** [loud noise]. | | neutral |
| 0:00 | “Ouch!”  E **puts down** the clipboard and **holds** his/her left thumb.  E **rubs** and **blows** his/her left thumb. | painful/ distressed |
| 0:10 | “I pinched my finger!”  E **rubs** and **blows** his/her left thumb. |  |
| 0:20 | “Ouch, my finger does really hurt.”  E **rubs** and **blows** his/her left thumb. |  |
| 0:30 | “But I think it’s getting better now.”  E **continues rubbing and blowing** his/her left thumb, but **gradually lower intensity of pain** display. | gradually less painful/ distressed |
| 0:40 | “Now it’s getting better again.”  The **pain slowly ceases**. |  |
| 0:50 | “Yes, the pain is almost gone now.”  The **pain slowly ceases**. |  |
| 0:60 | “That really hurt but I’m feeling better now. So let’s move on with the next game! [introduce the next task]” | neutral |

**Stick to the timing, even if the child reacts!**

**Looking behavior:** While making verbal pain expressions („That hurt so much”, “I pinched my finger”), E keeps eye contact with the child. Otherwise, E looks at his/her left thumb or on the floor. This ensures that the pain reaction is realistic and adequately involves the child (Zahn-Waxler et al., 1992 did not have eye contact at all. We decided against this as it seems unrealistic).

What if…

- … the child tells you something, e.g., when they last hurt their finger, or offers help, e.g. “I can sing you a song!” or “Shall I hand you a band-aid?”
  - Do not react to it, but stick to the instructions above.
- … the child asks something, e.g.: “What happened?”, “Are you feeling better now?”, [something unrelated]
  - Do not answer, but stick to the script instructions.
- … the child actively helps, e.g. hands a band aid

**B) Coding scheme for pain simulation task**

| **Rating** | **Hypothesis testing** | **Empathic Concern** | **Prosocial acts** | **Self-distress (for details, see next table)** | **Avoidance** |
| --- | --- | --- | --- | --- | --- |
| 0 | No gaze at victim Only looking at victim’s face or only looking at injury; only looking once at face and then at finger | No empathic concern **Slight** **change in facial expression** (Brow furrow, slight or fleeting changes), brief, one very slight movement (might be out of surprise and not concern) | No prosocial acts | No signs of distress | No signs of avoidance |
| 1 | Mulitple times looking from injury to victim’s face (exploratively scanning) | Empathic facial expression (persistent or repeated expression of concern that includes brow furrow, **overall more than 3 seconds**); **and / or** single empathic vocalization (uh, oh or ah) - *tone of voice* is decisive, not content;  **Or** single empahthic verbal utterance without change of facial expression | Briefly assisting verbally or nonverbally (pointing to/out helpful items);  Verbal elaboration (Be careful);  Non-egocentric statement (Mum has band aids);  Blaming the object;  Sharing own experiences with pain;  Empathic smile (eye-contact with E) in combination with approaching E (intention to cheer up E should be clear);  Verbally offering instrumental help (I can open the clipboard, Should I open the clipboard?) | Light signs of distress | Slight disengagement or avoidance of experimenter’s distress - averting the gaze, looks or turns away  Do not code if it is slight disengagement within last 10 seconds of PainSim. |
| 2 | Simple verbal exploration: Single verbal utterances ("Hurt?; Are you okay?);  Imitation (Saying "ow“, rubbing finger); Verbal sign of understanding (Saying "Yes" or "Mhm" immediatly after a cue); *content* is decisive;  **Or** postural approach with non-verbal exploration (pointing, focusing, head movement) | Moderate to more intense facial expression and either empathic verbal utterance ("I’m sorry"),  **Or** multiple vocalizations (soothing voice),  **Or** moderate gestural-postural expression (leaning forward/ pointing/head movement in combination with empathic face and/or tone);  expressed for a long duration, but with moderate intensity; OR: expressed intensely but relatively briefly | Verbally offering help (Can I help you? Do you need a band aid?);  Egocentric ("I") statement or question containing "you" ; Sharing (giving sth. to victim),  Start joint activitiy, etc. to cheer up victim (intention to cheer up E must be clear rather than ignore the incident, sign for joint activity: empathic or engaging tone) Protecting victim (placing object out of reach of E to prevent future injuries); Instrumental helping (opening the clipboard, showing how to open it) | Moderate signs of distress | Moderate disengagement: remains turned away a good portion of the episode (at least one-third, i.e., 20 seconds in sum) **Or** Occupation with own garment/accessory  Bury/hide face in hands/soft toy |
| 3 | Single incidence of combination of nonverbal and verbal exploration;  **Or** repeated verbal one-worded utterances;  **Or** single verbal exploration and sophisticated non-verbal exploration (longer postural approach to explore) | Great concern:  Substantial change in facial expression (expressed in cooing or sympathetic vocal tones, sympathy face);  **Or** moderate facial expression and substantial gestural-postural expression;  And each of those *needs to be combined with verbal utterances or vocalization as defined in 2* Expressed for long duration (at least 5 seconds) and with high intensity | Physical comforting (patting, hugging, kissing); getting helpful items (cool pad, band aid); requesting help from another adult; singing a song | Strong signs of distress | Strong disengagement: Child fully retreats from experimenter distress **Or** Distraction by attention to stamps / treasure map / sticker / drink (often associated with statements / questions about it as well as actions with it). *If it is happening with empathic concern or in a very happy tone, it could also be a* ***distraction*** *of the person in pain -> PSB* |
| 4 | Repeated, sophisticated attempts to comprehend the problem (e.g., asking, “Does it hurt? Did you pinch it?” **Or** looking behind or underneath the injury to ascertain cause) |  |  |  | Never engaged: Child is unresponsive - did not look at the victim at all throughout the entire episode  **Or** Leaving the room without explanation |

**Pain simulation coding scheme: Details of self-distress scale**

| **Score** | **Behavior** |
| --- | --- |
| 0 | No signs of distress |
| 1 | Light signs of distress in **posture** - Freezing (freezing in movement, remaining in one posture, rigid head position); Hunched shoulders |
|  | Light signs of distress in **facial expression**: - Open mouth; Movements/grimaces with the mouth (e.g. chewing on the upper/lower lip) |
|  | Light **self soothing behavior** - Suck thumb / Put finger to/in mouth, pick nose - Repetitive behavior (repeatedly touching/scratching/rubbing on face/arm/leg); tapping fingers/hands on/against table/chair). |
|  | Light signs of distress in **language** - Child stops talking in mid-sentence (could be surprise); This does not count as self-distress alone. |
|  | **Relief** after the end of the simulation; not to be coded if it occurs after empathic concern - relieved exhalation; Smile emerges/returns; Solidification is dissolved |
| 2 | Moderate signs of distress in **posture** - (Evasive/ embarrassing) movements because situation is uncomfortable (rocking back and forth on chair, squirming on chair, putting head back on neck) |
|  | Moderate signs of distress in **facial expression** - Anxious (raised eyebrows, open eyes, slightly downturned corners of mouth or open mouth, slightly rigid facial expression). - depressed facial expression (strongly contracted eyebrows (more/deeper than in empathic concern, often gaze lowered at the same time) |
| 3 | Strong signs of distress in **facial expression**: - Almost starts to cry (facial expression as if the child is about to start crying (in a moment), but still without tears and sounds; - Narrowed eyes, "sad" eyebrows, corners of mouth down). - Starts to cry |
|  | Strong signs of distress in **posture** - Backs off strongly - Leaving room while crying |

**C) Procedure of the puppet interview to assess the moral self-concept (MSC).**

Look, these characters are very curious and they want to know a few things from you. The characters always tell you something about themselves and then they ask you what you think about it. Let’s see what they say.

| **LEFT CHARACTER** | | | **RIGHT CHARACTER** | | | # |
| --- | --- | --- | --- | --- | --- | --- |
| I do like to play with the ball. | | | I **do not** like to play with the ball. | | | 0 |
| A lot 5 | A bit 4 | Middle 3 | | A bit 2 | A lot 1 |  |
| Sh_1 I like to share my pencils. | | | I **do not** like to share my pencils. | | | 1 |
| A lot 5 | A bit 4 | Middle 3 | | A bit 2 | A lot 1 |  |
| He_1 I **do not** like to help find something when another child cannot find it. | | | I like to help find something when another child cannot find it. | | | 2 |
| A lot 1 | A bit 2 | Middle 3 | | A bit 4 | A lot 5 |  |
| Co_1 I like to comfort a child, even if it was mean to me once. | | | I **do not** like to comfort a child, even if it was mean to me once. | | | 3 |
| A lot 5 | A bit 4 | Middle 3 | | A bit 2 | A lot 1 |  |
| He_2 I like to help another child clean up toys. | | | I **do not** like to help another child clean up toys. | | | 4 |
| A lot 5 | A bit 4 | Middle 3 | | A bit 2 | A lot 1 |  |
| He_3 I like to help pick something up again when another child drops something. | | | I **do not** like to help pick something up again when another child drops something. | | | 5 |
| A lot 5 | A bit 4 | Middle 3 | | A bit 2 | A lot 1 |  |
| Co_2 I **do not** stop playing my favourite game to comfort a crying child. | | | I stop playing my favourite game to comfort a crying child. | | | 6 |
| A lot 1 | A bit 2 | Middle 3 | | A bit 4 | A lot 5 |  |
| Co_3 I **do not** comfort a child, even when they have started the fight themselves. | | | I comfort a child, even when they have started the fight themselves. | | | 7 |
| A lot 1 | A bit 2 | Middle 3 | | A bit 4 | A lot 5 |  |
| Sh_2 I **do not** take care that everyone gets the same amount. | | | I take care that everyone gets the same amount. | | | 8 |
| A lot 1 | A bit 2 | Middle 3 | | A bit 4 | A lot 5 |  |
| Sh_3 I **do not** like to let other children play with my toys. | | | I like to let other children play with my toys. | | | 9 |
| A lot 1 | A bit 2 | Middle 3 | | A bit 4 | A lot 5 |  |

*Blue highlighted statement is uttered first

Keep intonation identical between affirmative and negative statement

**D) Descriptive results**

**Table 1**

Descriptive statistics of all variables

| **Variable** | **Mean** | **SD** | **Min** | **Max** |
| --- | --- | --- | --- | --- |
| Pain sim: Hypothesis testing | 1.69 | 1.02 | 0 | 4 |
| Pain sim: Empathic concern | 1.11 | 0.96 | 0 | 3 |
| Pain sim: Prosocial acts | 0.55 | 0.75 | 0 | 3 |
| Pain sim: Self-distress | 0.67 | 0.65 | 0 | 3 |
| Pain sim: Avoidance | 0.76 | 1.12 | 0 | 4 |
| Theory of Mind | 3.21 | 1.42 | 0 | 5 |
| Emotion comprehension | 3.18 | 1.77 | 0 | 8 |
| Moral self-concept comforting | 3.68 | 1.16 | 1 | 5 |
| Accuracy in Flanker task | 0.87 | 0.20 | 0 | 1 |
| Surgency | 4.62 | 0.87 | 1.83 | 6.67 |
| Age in months | 60.65 | 11.43 | 42 | 83 |

**Table 2**

# Correlation matrix of all variables (using the imputed data set)

|  | (1) | (2) | (3) | (4) | (5) | (6) | (7) | (8) | (9) | (10) | (11) |
| --- | --- | --- | --- | --- | --- | --- | --- | --- | --- | --- | --- |
| Pain sim: Hypothesis testing (1) | 1 |  |  |  |  |  |  |  |  |  |  |
| Pain sim: Empathic concern (2) | .52*** | 1 |  |  |  |  |  |  |  |  |  |
| Pain sim: Prosocial acts (3) | .34*** | .56*** | 1 |  |  |  |  |  |  |  |  |
| Pain sim: Self-distress (4) | -.29*** | -.43*** | -.41*** | 1 |  |  |  |  |  |  |  |
| Pain sim: Uninvolvement (5) | -.07 | -.27*** | -.16** | -.01 | 1 |  |  |  |  |  |  |
| Theory of Mind (6) | -.02 | .05 | .05 | -.07 | .02 | 1 |  |  |  |  |  |
| Emotion comprehension (7) | .12* | .08 | .16** | -.06 | -.01 | .29*** | 1 |  |  |  |  |
| Moral self-concept (8) | .02 | .02 | .15** | -.11* | -.05 | .06 | .16** | 1 |  |  |  |
| Accuracy in Flanker task (9) | -.01 | .07 | .12* | -.15** | -.06 | .32*** | .12* | .13** | 1 |  |  |
| Surgency (10) | .14** | .19*** | .17** | -.11* | .07 | .08 | .03 | .03 | .01 | 1 |  |
| Age in months (11) | .20*** | .09 | .19*** | -.19*** | .06 | .38*** | .54*** | .19*** | .38*** | .04 | 1 |

*Note:* ***p < .001; **p < .01; *p < .05

**Table 3**

# Partial correlation matrix, controlling for age (using the imputed data set)

|  | (1) | (2) | (3) | (4) | (5) | (6) | (7) | (8) | (9) | (10) |
| --- | --- | --- | --- | --- | --- | --- | --- | --- | --- | --- |
| Pain sim: Hypothesis testing (1) | 1 |  |  |  |  |  |  |  |  |  |
| Pain sim: Empathic concern (2) | .51*** | 1 |  |  |  |  |  |  |  |  |
| Pain sim: Prosocial acts (3) | .31*** | .56*** | 1 |  |  |  |  |  |  |  |
| Pain sim: Self-distress (4) | -.26*** | -.42*** | -.39*** | 1 |  |  |  |  |  |  |
| Pain sim: Uninvolvement (5) | -.08 | -.28*** | -.17** | .00 | 1 |  |  |  |  |  |
| Theory of Mind (6) | -.11* | .02 | -.03 | .01 | .00 | 1 |  |  |  |  |
| Emotion comprehension (7) | -.02 | .04 | .07 | .05 | -.05 | .10 | 1 |  |  |  |
| Moral self-concept (8) | -.02 | .00 | .12* | -.08 | -.07 | -.01 | .07 | 1 |  |  |
| Accuracy in Flanker task (9) | -.10 | .04 | .05 | -.08 | -.09 | .20*** | -.11* | .06 | 1 |  |
| Surgency (10) | .14* | .19*** | .17** | .10* | .06 | .07 | .01 | -.02 | -.01 | 1 |

*Note:* ****p* < .001; ***p* < .01; **p* < .0

**Table 4**

Pairwise comparisons of pain simulation variables across empathy-related profiles

| **Variable** | **Prosocial vs Uninvolved** | **Prosocial vs Self-distressed** | **Prosocial vs Empathic** | **Uninvolved vs Self-distressed** | **Uninvolved vs Empathic** | **Self-distressed vs Empathic** |
| --- | --- | --- | --- | --- | --- | --- |
| Hypothesis Testing | Prosocial > Uninvolved*** | Prosocial > Self-distressed*** | n.s. | Uninvolved > Self-distressed* | Uninvolved < Empathic*** | Self-distressed < Empathic*** |
| Empathic Concern | Prosocial > Uninvolved*** | Prosocial > Self-distressed*** | n.s. | n.s. | Uninvolved < Empathic*** | Self-distressed < Empathic*** |
| Prosocial Acts | Prosocial > Uninvolved*** | Prosocial > Self-distressed*** | Prosocial > Empathic*** | Uninvolved > Self-distressed*** | Uninvolved < Empathic*** | Self-distressed < Empathic*** |
| Self-Distress | Prosocial < Uninvolved** | Prosocial < Self-distressed*** | n.s. | Uninvolved < Self-distressed*** | Uninvolved > Empathic** | Self-distressed > Empathic*** |
| Avoidance | Prosocial < Uninvolved*** | n.s. | n.s. | Uninvolved > Self-distressed*** | Uninvolved > Empathic*** | n.s. |

***Note:*** Results from Tukey’s HSD post-hoc tests following significant ANOVAs. Direction of difference indicated by inequality symbols (e.g., Prosocial > Uninvolved indicates Prosocial profile scored significantly higher than Uninvolved profile). ****p* < .001, ***p* < .01, **p* < .05, n.s. = not significant.
